# The effect of atmospheric pressure cold plasma on the inactivation of *Escherichia coli* in sour cherry juice and its qualitative properties

**DOI:** 10.1002/fsn3.1364

**Published:** 2020-01-20

**Authors:** Seyed Mehdi Hosseini, Sajad Rostami, Bahram Hosseinzadeh Samani, Zahra Lorigooini

**Affiliations:** ^1^ Department of Mechanical Engineering of Biosystems Shahrekord University Shahrekord Iran; ^2^ Medical Plants Research Center Basic Health Sciences Institute Shahrekord University of Medical Sciences Shahrekord Iran

**Keywords:** cold plasma, *Escherichia coli*, nonthermal method, response surface method, sour cherry juice

## Abstract

One of the nonthermal methods is the atmospheric pressure cold plasma (APCP). In this study, the effect of cold plasma on the reduction of *Escherichia coli* bacteria and qualitative properties of sour cherry juice, including total phenolic content (TPC), total anthocyanin content (TAC), and vitamin C, were investigated. Independent variables included plasma exposure time (1, 5, and 9 min), applied field intensity (25, 37.5, and 50 kV/cm), feeding gas oxygen content (0%, 0.5%, and 1%), and sample depth (0.5, 1, and 1.5 cm). The results show that increased oxygen content in argon has the greatest effect on the reduction of bacteria, and plasma exposure decreased 6 logarithmic periods of *E. coli* bacteria in sour cherry juice. Optimization results showed when all bacteria were eliminated by plasma, TPC remained unchanged, and TAC and vitamin C decreased by 4% and 21%, respectively, while thermal methods increased TPC by 23% and decreased TAC and vitamin C by 26% and 77%, respectively. These results indicate that, compared with conventional thermal methods, sour cherry juice pasteurization using APCP has little effect on the juice qualitative properties, and this method can serve as a suitable alternative to conventional thermal methods.

## Graphical abstract

These results indicate that, compared with conventional thermal methods, sour cherry juice pasteurization using atmospheric pressure cold plasma has little effect on the juice qualitative properties, and this method can serve as a suitable alternative to conventional thermal methods.

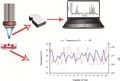

## 1 INTRODUCTION

Diseases caused by contaminated agricultural products and foodstuffs as a concern have drawn many researchers' attention (Niemira, 2012). In order to reduce these diseases, pasteurization of foodstuffs is essential. Today, one of the most important methods of decontaminating is thermal pasteurization. These methods have a great influence on the deactivation of microorganisms, but high‐temperature exposure in these methods causes undesirable changes in the taste, color, smell, texture, and appearance properties of the materials (Mohamed & Eissa, 2012; Samani, Lorigooini, et al., 2018). Besides that, the demand of consumers for the use of fresh and minimally processed foodstuffs has led to the many researchers' directing attention to new decontamination methods that preserve the qualitative properties of the materials (Adekunte, Tiwari, Cullen, Scannell, & O'Donnell, 2010; Hou et al., 2019; Mohamed & Eissa, 2012).

Atmospheric pressure cold plasma (APCP) is one of the nonthermal methods for food decontamination that can be used to treat materials under room temperature and atmospheric pressure (Gao, Zhuang, Yeh, Bowker, & Zhang, 2019; Niemira, 2012). Plasma is an ionized gas in which free electrons are approximately equal to the number of positive ions (Fridman & Kennedy, 2004). Cold plasma can affect a wide range of microorganisms so that the use of this method has been widely considered (Shi et al., 2011). Several studies have been conducted on the use of APCP to reduce bacteria in solids, including freshly cut apple skin (Segura‐Ponce, Reyes, Troncoso‐Contreras, & Valenzuela‐Tapia, 2018), biofilms infected with *Escherichia coli* (Niemira, Boyd, & Sites, 2018), eggshell (Harouni & Abbasi, 2013; Ragni et al., 2010), chicken skin and muscle (Noriega, Shama, Laca, Díaz, & Kong, 2011), and cheese pieces (Song et al., 2009).

In this regard, researchers studied the reduction of *E. coli* in raw milk by using cold plasma (Coutinho et al., 2019; Gurol, Ekinci, Aslan, & Korachi, 2012). In another study, the effects of cold plasma on the inactivation of three types of bacteria including *Staphylococcus aureus*, *E. coli*, and *Candida albicans* in orange juice and its qualitative properties were investigated (Shi et al., 2011). Investigations have shown that the use of the APCP in reducing the microbial load of liquid food has been in cases that the low volumes and low depths of liquids were tested (Gurol et al., 2012; Shi et al., 2011; Surowsky, Fröhling, Gottschalk, Schlüter, & Knorr, 2014) and research on the inactivation of bacteria in greater depths of liquids seems necessary.

Since the sour cherry with *Prunus cerasus* L. scientific name is one of the most important horticultural products across the world, about 85 percent of this product is used in a processed form such as juice, jam, and etc. (Toydemir et al., 2013). The aim of this study was to investigate the effects of the DBD‐APCP on the inactivation of *E. coli* in sour cherry juice and its qualitative properties. Then, the results were compared with the results of the conventional thermal method.

## 2 MATERIALS AND METHODS

### 2.1 Generation of cold plasma

**Figure 1:**
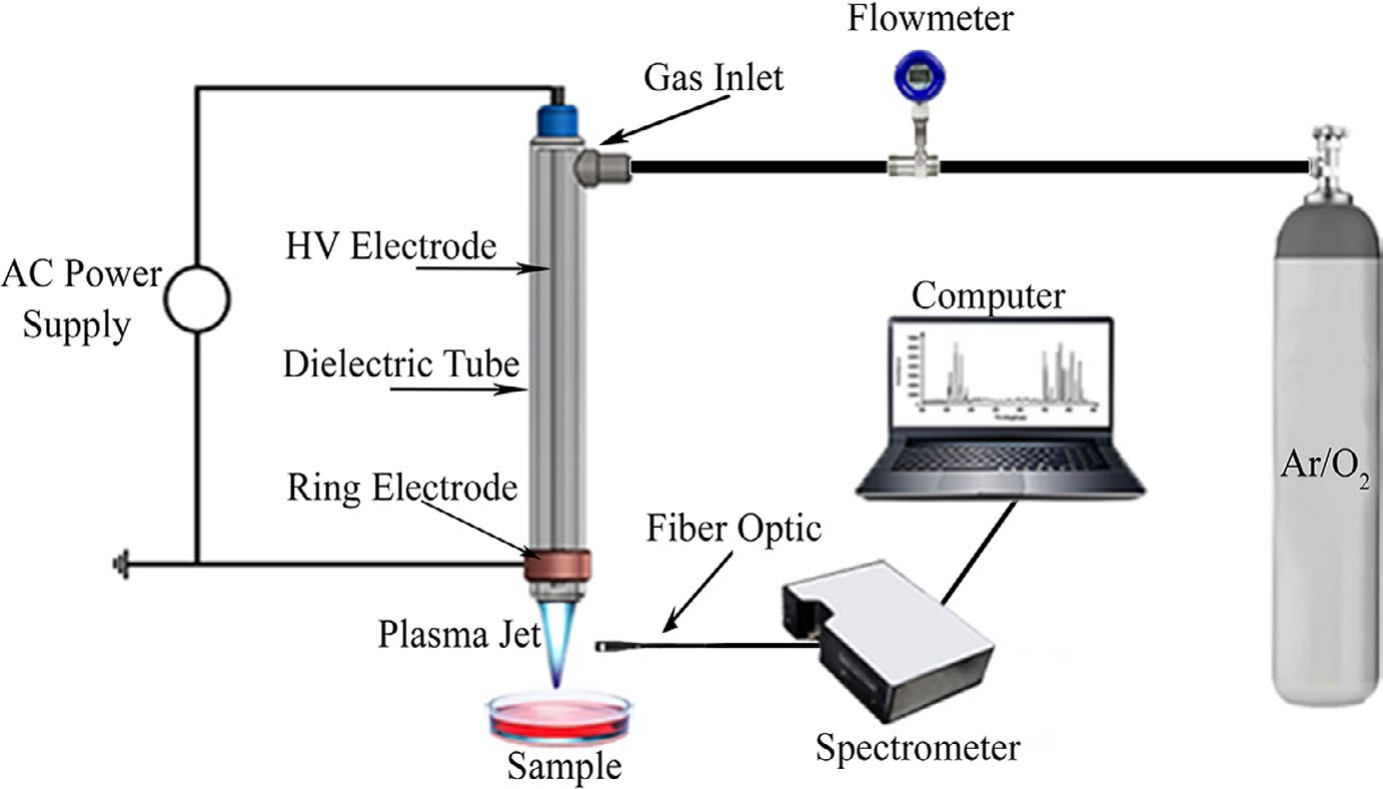
The schematic illustration of the plasma generation system

In this study, dielectric barrier discharge (DBD) was used to generate plasma. The geometry of the plasma generation system is very flexible in the DBD method (Misra, Schlüter, & Cullen, 2016). In this study, coaxial electrodes were used to create a plasma generation system. The fabricated nozzle includes a ceramic tube dielectric, a copper ring electrode, and a tungsten central electrode (Figure 1), and these electrodes were connected to an AC power supply. The output voltage of the power supply varied between 0 and 20 kV, and the frequency was set at 20 kHz. The diameter of the central electrode 1 mm, the gap between the central electrode and the dielectric 4 mm, the internal radius and length of the ring electrode 5.5 and 10 mm, respectively, the thickness, internal radius, and length of the dielectric are 1 mm, 4.5 mm, and 10 cm, respectively, and the feeding gas flow 5 slm (standard liter per minute) were considered (Gas flow was measured and controlled by a flowmeter), and the combination of argon and oxygen was used as feeding gas (Surowsky et al., 2014). Samples were placed in circular glass plates of 2 cm in diameter. The distance between the surface of the samples and the nozzle was adjustable and was set at 2 cm for all experiments. Figure 1 shows the schematic illustration of the plasma generation system designed in this study (Video S1). Plasma properties were determined by OES and temperature measurement. In order to identify the plasma species, the emitted spectra were recorded by using a spectrophotometer (Ocean Optic, HR2000+CG) in the wavelength range of 200–900 nm (Figure 1). For this purpose, the detector was placed perpendicularly to the nozzle axis at a distance of 2 cm from the nozzle. Then, the data obtained through the spectroscopy were analyzed by using the “AvaSoft” software version 8 and the related diagrams are plotted, and the peak locations were compared with the NIST atomic spectra database to identify the particles generated from the plasma (Huang, Yu, Hsieh, & Duan, 2007; Surowsky et al., 2014). Plasma coldness was also determined by measuring the temperature of the samples. Temperature measurements were performed for all samples before and after plasma exposure (Surowsky et al., 2014).

### 2.2 Preparation of microbial samples and microbial tests

For this purpose, a certain amount of sour cherry fruit of Mashhad Champa variety was purchased from local markets. First, the fruits were washed, cleaned, and cored. The prepared fruits were then dewatered using an electric juicer. In order to separate pulp suspensions and tissue components, the extracted juice was poured into a centrifuge with the speed of 6,000 rpm (4,307 *g*) for 20 min. For complete separation of the remaining suspended particles, the transparent portion of the extract was passed through a Whatman filter paper using a vacuum pump (Hosseinzadeh Samani, Minaee, & Khoshtaghaza, 2015). The samples were then sterilized in autoclaved glass containers and sterilized using an autoclave at 121°C, under 15 psi for 15 min. Next, the sour cherry juice samples were contaminated with *E. coli* bacteria to determine the reduction rate of bacteria in each sample after undergoing the process of interest. To do this, a few colonies of *E. coli* bacteria cultured 24 hr ago were transferred into a sterile test tube containing physiological serum (sodium chloride 9%). Then, the samples were contaminated by adding an appropriate volume of a solution prepared with autoclaved sour cherry juice (1:9 v/v). The bacteria was counted before and after the juice samples were treated by plasma. To count bacteria, standard (viable) plate count method was used, and for each sample, five dilutions were obtained, and from each dilution, 1 ml was cultured on MacConkey Agar culture medium (all of these steps were carried out under a hood). The prepared Petri dishes were then incubated for 24 hr at 37°C to encourage the growth of *E. coli* bacteria. Finally, the number of colonies per plate was determined and the bacteria in each sample were counted (Deng, Cheng, Ni, Meng, & Chen, 2010).

### 2.3 Evaluation of chemical and qualitative properties of sour cherry juice

In the next step, the chemical and qualitative properties of the sour cherry juice were evaluated by measuring the total phenolic content (TPC), total anthocyanin content (TAC), and vitamin C. It should be noted that the unsterilized sample was used for plasma treatment and chemical evaluation.

#### 2.3.1 Measurement of TPC

Total phenolic content was measured by using the Folin–Ciocalteu colorimetric method. For this purpose, a volume equivalent to 0.0025 g of the sour cherry juice was mixed with 1 ml of methanol. The volume of the resulting mixture was increased to 2.25 ml by addition of distilled water. Then, 0.25 ml of Folin–Ciocalteu (10% v/v) was added to the mixture and shaken to mix thoroughly. After 5 min, 2 ml of sodium carbonate “Na_2_CO_3_ (20% v/v)” was added. The samples were placed in the dark at 25°C for 2 hr, and then, the absorbance was read at a wavelength of 765 nm by using a spectrophotometer (UV‐1800). By placing the results obtained from the absorbance of the samples in the calibration curve, the TPCs of the samples were measured and expressed as mg of gallic acid equivalent (GAE) in 100 g of the sour cherry juice. All experiments were performed in triplicate. To plot the standard curve, various dilutions of gallic acid were prepared and the standard curve is drawn (Liang, Yue, & Li, 2010).

#### 2.3.2 Measurement of TAC

The TACs of samples were measured by using the pH differential method and based on cyanidin‐3‐glucoside as dominant anthocyanin of sour cherry (Blando, Gerardi, & Nicoletti, 2004). To this end, two buffers, “potassium chloride (KCL, pH = 1)” and “sodium acetate (HCL, pH = 4.5)” were used. For each experiment, 2 ml of each sample was poured into two 25‐ml volumetric flask, and then, one of the balloons was reached to volume by the KCL buffer and the other one was reached to volume by adding HCL buffer. After 20–40 min, at wavelengths of 520 and 700 nm, the absorbance was read by the UV‐1800 spectrophotometer two times, once with the KCL buffer and again with the HCL buffer, and the TAC was measured by Equation 1.

(1) $$\text{TAC}\;\left(\text{mg C}3\text{GE}/L\right)=\frac{A\times \;\text{Mw}\;\times \;\text{DF}\;\times 10^{3}}{\varepsilon \times L}$$

where Mw is molar mass of cyanidin‐3‐glucosides, *ε*: molar extinction coefficient for cyaniding‐3‐glucoside, DF: dilution factor and *L*: cuvette optical path length, and *A*: absorbance, which was calculated by Equation 2.

(2) $$A={\left(A_{520}-A_{700}\right)}_{\text{pH}=1}-{\left(A_{520}-A_{700}\right)}_{\text{pH}=4.5}$$

where A_520_ is absorbance at 520 nm and A_700_: absorbance at 700 nm wavelength (Lee, Durst, & Wrolstad, 2005; Rouhani, Eyenafshar, & Ahmadzadeh, 2015). All experiments were carried out in triplicate.

#### 2.3.3 Determination of vitamin C

Concentration of vitamin C was determined used by a redox titration using iodine (Anonymous, 2018). At first, 20 ml aliquot of the sample into a 250‐ml conical flask pipetted and added about 150 ml of was distilled water and 1 ml of starch (0.05% m/v) indicator solution. The sample was titrated with 0.005 mol/L iodine solution. The endpoint of the titration was identified as the first permanent trace of a dark blue‐black color due to the starch–iodine complex. The average volume of iodine solution was calculated from concordant titers. The moles of iodine were calculated by the reacting titration Equation 3, so the number of moles of ascorbic acid reacted was determined. The concentration was calculated, in mg/100 ml of ascorbic acid, in the sample. All experiments were performed in triplicate.

(3) $$\text{Ascorbic acid}+I_{2}\to 2\;I^{-}+\text{dehydroascorbic acid}$$

In Equation 3, I^−^ is iodide ions and I_2_ is iodine (Anonymous, 2018).

### 2.4 Thermal pasteurization methods

First, the 5 ml of sour cherry juice samples were pasteurized by a conventional thermal method at a temperature range of 85°C for 5 min (Samani, Lorigooini, et al., 2018). Then, TPC, TAC, and vitamin C level were determined and the results were compared with the results obtained through the APCP method.

### 2.5 Analysis, modeling, and optimization of experiments

In this study, the effects of DBD‐APCP on the inactivation of *E. coli* in the sour cherry juice and its qualitative properties were investigated. To design the experiments, modeling, and optimization of the response surface method (RSM), results were used. In the RSM, in order to obtain the equation of the model and to determine the target function for optimization, regression Equation 4 must be solved:

(4) $$Y_{i}=\beta_{0}+\sum \beta_{i}x_{i}+\sum \beta_{\mathrm{ij}}x_{i}x_{j}+\sum \beta_{\mathrm{jj}}x_{i}^{2}+\varepsilon$$

where, *β*_0_, *β_i_*, *β_ij_*, and *β_jj_* are constant coefficients, *x_i_* and *x_j_* independent variables of the process and *ε* random error (Behruzian, Hosseinzadeh Samani, Rostami, Lorigooini, & Behruzian, 2018; Samani, Gudarzi, et al., 2018).

In this study, independent variables were plasma exposure time (min), the depth of samples (cm), the average field strength created at the distance between the electrons (in this article referred to as field strength) (kV/cm), and the oxygen content in argon (%), whose effects of them on the inactivation of *E. coli* bacteria, and the levels of TPC, TAC, and vitamin C in sour cherry juice were studied. The range of changes in independent variables in the experiments is shown in Table 1 (encoded at three levels). Under these conditions, 30 different tests were designed.

**Table 1:** Levels of independent variables selected for the design of experiments

Independent variable	Range of level		
	−1	0	1
Plasma exposure Time (min)	1	5	9
Field strength (kV/cm)	25	37.5	50
Depth (cm)	0.5	1	1.5
Oxygen in argon (%)	0	0.5	1

## 3 RESULTS AND DISCUSSION

### 3.1 Characteristics of the plasma source

**Figure 2:**
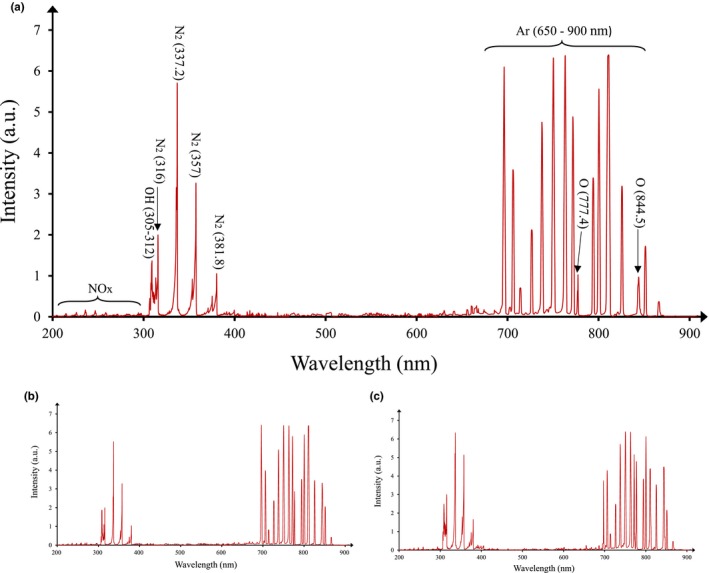
Comparison of the emission spectrum of APCP from (a) argon (b) 99.5% argon and 0.5% oxygen and (c) 99% argon and 1% oxygen

The curves of the emission spectra, for pure argon, 99.5% argon and 0.5% oxygen, and 99% argon and 1% oxygen, are shown in Figure 2 The intensity of the lines corresponding to the argon atoms in the range of 650–900 nm is high in all curves (Huang et al., 2007; Mohamed, Al Shariff, Ouf, & Benghanem, 2016). In this range, the lines corresponding to the oxygen atoms at 777.4 and 844.5 nm wavelengths are also observed. Also, the reaction of free electrons with N_2_ and O_2_ molecules results in the formation of excited N_2_ molecules, $N_{2}^{+}$, N^+^, O_3_, and a little of NOx in the UV‐C range. Due to the reaction of reactive species obtained from the plasma with water in the air and sour cherry juice, OH and O radicals are produced (Huang et al., 2007; Mohamed et al., 2016). Figure 2 shows the concentrations of O, OH, and O_3_ radicals, when feeding gas contains 0.5% and 1% oxygen, compared to the state that feeding gas is pure argon, will increase by 205%, 38%, 16%, and 359%, 83%, and 34%, respectively.

**Figure 3:**
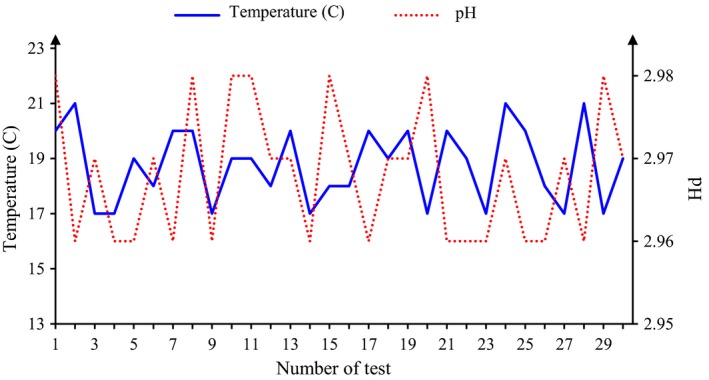
Changes in the temperature and pH in tests

In this study, the nonthermally of the generated plasma was determined by measuring the temperature of the samples. Before the tests, the temperature of all samples was about 21°C, and after plasma exposure, the temperature varied from 17 to 21°C depending on the test conditions. By increasing the plasma exposure time and reducing the depth, the temperature of the samples was further reduced. Reducing the temperature at higher field strengths is less due to the higher temperature of the plasma jet. Figure 3 shows the changes in temperature and pH in the samples after plasma exposure. The reduction of temperature is due to the lower temperature of the feeding gas than the samples (Surowsky et al., 2014).

### 3.2 The effect of plasma on the inactivation of *E. coli* bacteria

The ANOVA results show the effects of plasma exposure time, depth of samples, field strength, and oxygen content in argon, as well as the effects of time × field strength, time × oxygen percentage in argon, depth of samples × oxygen percentage in argon and field strength × oxygen percentage on the inactivation of the *E. coli* bacteria in sour cherry juice are significant (*p* < .01; Table 2). The total sum of squares obtained from ANOVA shows that, among the main variables, the percentage of oxygen in argon accounts for 67.5% of variance in data has the greatest effect on the inactivation of bacteria, followed by field strength, plasma exposure time, and depth of sample, explaining 12%, 6.8%, and 4.3%, of variance in the data, respectively.

**Table 2:** The analysis of variance results for the effects of plasma exposure time, depth of samples, field strength, and oxygen percentage in argon on the inactivation of *Escherichia coli* bacteria in sour cherry juice

Source	*df*	Sum of squares	Mean square
Model	14	62.99	4.50**
Time (min)	1	4.27	4.27**
Depth (cm)	1	2.98	2.98**
Field strength (kV/cm)	1	7.55	7.55**
Oxygen in argon (%)	1	42.57	42.57**
Time × depth	1	0.038	0.038^ns^
Time × field strength	1	0.67	0.67**
Time × oxygen in argon	1	0.52	0.52**
Depth × field strength	1	3.630E‐003	3.630E‐003^ns^
Depth × oxygen in argon	1	0.81	0.81**
Field strength × oxygen in argon	1	0.80	0.80**
Time^2^	1	0.17	0.17^ns^
Depth^2^	1	0.058	0.058 ^ns^
Field strength^2^	1	0.35	0.35^ns^
Oxygen in argon^2^	1	0.089	0.089^ns^
Residual	15	0.90	0.060
Lack of fit	10	0.74	0.074^ns^
Pure error	5	0.16	0.032
Cor total	29	63.89	

Note

^ns^Not significant.

** Significant effect at 1% level.

**Figure 4:**
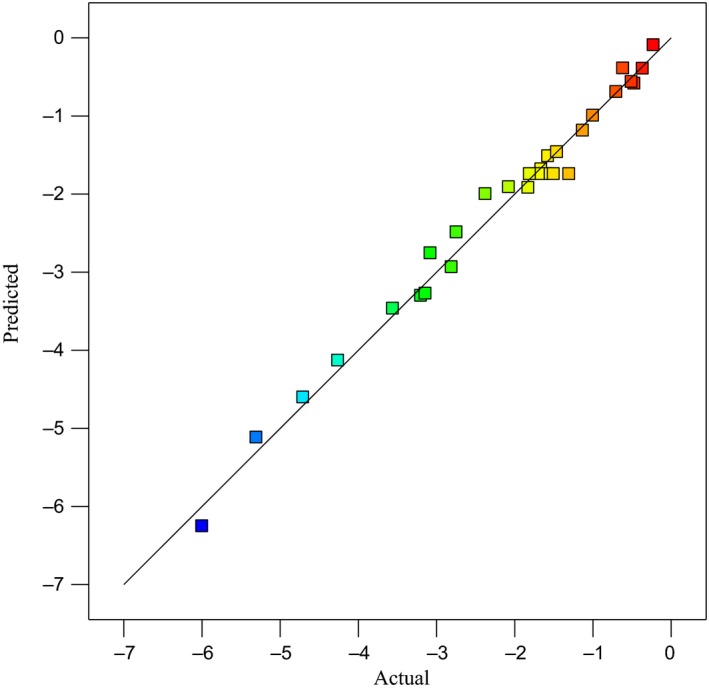
Verification of the actual data versus the data obtained from the model

The obtained model from the RSM to determine the rate of reduction in *E. coli* bacteria in the sour cherry juice is a fully quadratic model with an explanation coefficient of 0.985 and a standard error of 0.25. Figure 4 shows the variance in actual data versus variance in the data obtained from the model, and Equation 5 represents the obtained model in the encoded condition and Equation 6 represents the actual model.

(5) $$\begin{array}{c} \text{Log}\;\left(N/N_{0}\right)=1.75-0.49\times T+0.41\times D-0.65\times F-1.54\times \text{OC}+0.049\times T\times D\\ -0.020\times T\times F-0.18\times T\times \text{OC}-0.015\times D\times F+0.22\times D\times F\\ -0.22\times F\times \text{OC}+0.26\times {\left(T\right)}^{2}+0.15{\left(D\right)}^{2}-0.37{\left(F\right)}^{2}-0.19\times {\left(\text{OC}\right)}^{2} \end{array}$$

(6) $$\begin{array}{c} \text{Log}\;\left(N/N_{0}\right)=-3.18+0.26\times T-0.62\times D+0.17\times F-1.44\times \text{OC}-0.02\times T\times D\\ -0.004\times T\times F-0.09\times T\times \text{OC}-0.002\times D\times F+0.89\times D\times \text{OC}\\ -0.06\times F\times \text{OC}-0.02\times {\left(T\right)}^{2}+0.56\times {\left(D\right)}^{2}-0.003\times {\left(F\right)}^{2}-0.74\times {\left(\text{OC}\right)}^{2} \end{array}$$

where plasma exposure time (*T*, min), the depth of samples (*D*, cm), the average field strength created at the distance between the electrons (in this article referred to as field strength) (*F*, kV/cm), and the oxygen content in argon (OC, %).

In Equation 5, the coefficients of oxygen percentage in argon and field strength are the largest, which, as the sum of the squares obtained in Table 2 also shows, represents the greater effect of these two variables on bacterial inactivation compared to other variables. In Equation 5, the negative coefficient of selected variables indicates that with increasing each of them, the number of *E. coli* bacteria decreases.

**Figure 5:**
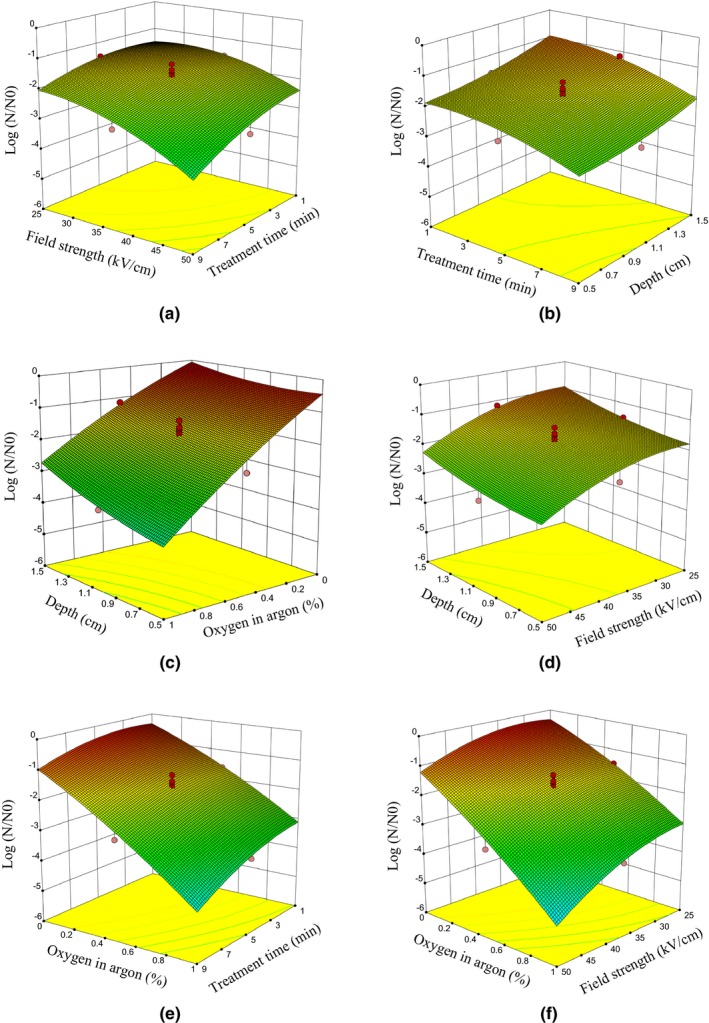
Effects of plasma exposure time, field strength, oxygen percentage in argon, and depth of samples on inactivation of *Escherichia coli* bacteria in sour cherry juice

Figure 5a,b,e shows that the number of residual *E. coli* bacteria in the sour cherry juice is a function of plasma exposure time, so that with increasing plasma exposure time, the number of damaged and destroyed bacteria increases (Fröhling et al., 2012; Niemira et al., 2018; Segura‐Ponce et al., 2018; Ziuzina, Patil, Cullen, Keener, & Bourke, 2014). In such a way, increasing plasma exposure time from 1 to 9 min resulted in a reduction of 75% of bacteria. In milk samples contaminated with *E*. *coli*, 54% of bacteria were eliminated after 3 min of plasma exposure (Gurol et al., 2012). Increasing exposure time from 1 to 8 min reduced 5 logarithmic cycles of *Citrobacter freundii* bacteria in apple juice (Surowsky et al., 2014).

As well as, according to Figure 5b,c,d the effect of APCP on the reduction of *E. coli* bacteria decreases with increasing the depth of the samples. This result shows the penetration of the plasma into the inner layers of the material is low, and with cold plasma method, only the surface layers of the material can be decontaminated, and plasma products such as ROS, active nitrogen species (RNS), ozone (O_3_), and UV can only penetrate a few micrometers (Misra et al., 2016). The results of the studies showed that the plasma could eliminate bacteria in biofilms to depths above 15 μm (Xiong, Du, Lu, Cao, & Pan, 2011) and 25 μm (Pei et al., 2012). It should be noted that the plasma composition and its flow rate influence the permeability. For example, hydrogen peroxide (H_2_O_2_) has a half‐life of 10 ms and is relatively stable whereas other oxygen species such as single oxygen (O) and hydroxyl radicals (OH) have a half‐life of 1 μs and 1 ns, which makes the penetration of peroxide hydrogen particles higher than the O and OH radicals.

Because the structure of the liquid foods is different from the solids and different layers of the liquids can be exposed to the plasma, and the liquids have water molecules that can react with ROS, the depth at which plasma can inactivate the bacteria is greater than the corresponding depth in solid materials (Misra et al., 2016). In the study, as shown in Figure 5b considering the two variables of the treatment time and the depth of the sour cherry juice samples, the highest inactivation rate of *E*. *coli* bacteria is observed in samples with a depth of 0.5 cm at the exposure time of 9 min (cross sections of all samples were identical).

The field strength applied in the region of generation plasma is directly correlated with the applied voltage (Misra et al., 2016). Therefore, in our study, changing the voltage of the power supply was used to change the field strength. Figure 5a,d,f and Equation 5 show that by increasing the field strength (increasing the applied voltage), more bacteria are deactivated. Thus, with an increase in the field strength from 25 to 50 kV/cm, disregarding other variables' effects, over 52% of the bacteria were damaged and eliminated. This result is similar to the results of some studies in which the effect of increasing the voltage and consequently field strength on bacterial inactivation has been investigated (Bermúdez‐Aguirre, Wemlinger, Pedrow, Barbosa‐Cánovas, & Garcia‐Perez, 2013; Tolouie, Mohammadifar, Ghomi, & Hashemi, 2017; Xiaohu, Feng, Ying, Jing, & Jianjun, 2013). This reduction can be attributed to increasing in feeding energy due to increased field strength and, consequently, increased the concentration of ROS derived from plasma (Lu, Patil, Keener, Cullen, & Bourke, 2014; Misra et al., 2016). Figure 5a shows that the effect of increasing field strength on the inactivation of bacteria in longer exposure times (over 5 min) is greater than that in shorter (<5 min) ones (Bermúdez‐Aguirre et al., 2013), because in this condition, the concentrations of plasma species at a certain interval are more than those when field strength is lower (Noriega et al., 2011).

In this study, the pure argon and the combination of argon and oxygen were used for plasma generation. The OES results (Figure 2) and ANOVA results (Table 2) and Figure 5c,e,f show that by adding oxygen to argon, the bacterial deactivation ability of the system increases (Deng et al., 2010; Gurol et al., 2012; Li, Sakai, Watanabe, Hotta, & Wachi, 2013; Uhm, Lim, & Li, 2007). Adding 2.14% oxygen to argon led to a change in the time of the reduction by five cycles of *E. coli* bacteria from 5 min to 30 s (Huang et al., 2007). Figure 5e shows in longer exposure times bacteria and eliminate them, the effect of increasing oxygen in reducing bacteria is greater than in shorter ones, because with increasing oxygen percentage, because by increasing time the concentration of ROS increases and the prolonged exposure time provides enough time for the particles to penetrate into bacteria and eliminate them (Huang et al., 2007; Surowsky et al., 2014).

Also, the obtained results from Table 2 and Figure 5f showed that the effect of increasing field strength on the inactivation of *E. coli* bacteria was more pronounced when the feeding gas contained a higher percentage of oxygen, because simultaneous increases in field strength and oxygen content produce high concentrations of ROS (Huang et al., 2007; Perni, Liu, Shama, & Kong, 2008). Therefore, in this study, the greatest reduction in *E. coli* bacteria occurred when plasma exposure time, the field strength, and oxygen percentage in argon were high (9 min, 50 kV/cm and 1%, respectively), and the depth of the sour cherry juice samples was minimum (0.5 cm). This value is equal to 6 logarithms cycles.

### 3.3 The mechanism of inactivation of *E. coli* in sour cherry juice by APCP

Temperature measurements showed the temperature of the samples in all experiments was <21°C (Figure 3). One of the other factors that can destroy the bacterial is the acidity of the samples under the experiment's conditions. In order to study this, the pH of the samples before and after the experiments was measured, and it was observed that the pH of the untreated samples was 2.99, and after the plasma exposure, the changes in sour cherry juice sample's pH, according to the experiment's conditions, were obtained 0.03 (Figure 3). The small effect of this change does not contribute greatly to reducing the sour cherry juice microbial load (Shi et al., 2011).

UV photons generated by the APCP can also be effective in destroying bacteria. The results of the OES showed that UV emission in this study (at wavelengths below 280 nm) was negligible. In addition, the researchers have argued the UV produced is rapidly weakened and does not reach the liquid surface. Therefore, UV emissions have little effect on the inactivation of *E*. *coli* bacteria in sour cherry juice (Deng, Shi, & Kong, 2006; Laroussi & Leipold, 2004; Shi et al., 2011).

According to studies on the destruction of bacteria by cold plasma, it can be argued that charged particles (e.g., positive and negative electrons) and ROS play the most important role in inactivating bacteria (Deng et al., 2010; Li et al., 2013; Mohamed et al., 2016). The reactions that cause the formation of ROS are shown in Equations (7), (8), (9), (10), (11) (Bruggeman et al., 2016; Misra et al., 2016; Shi et al., 2011):

(7) $$O_{2}+e\to O+O$$

(8) $$O+O_{2}+O_{2}\to O_{3}+O_{2}$$

(9) $$H_{2}O+e\to H+OH+e$$

(10) $$H_{2}O+O\to OH+OH$$

(11) $$H_{2}O+O_{3}\to OH+OH+O_{2}$$

Researchers have argued that ROSs created by APCP and charged particles can cause damage to bacteria by morphological changes in cell and change in the structure of cell membrane (Ali et al., 2014; Deng et al., 2006; Yang et al., 2011). The cell walls in gram‐negative bacteria such as *E. coli* are covered by a thin layer of peptidoglycan and an outer membrane of lipopolysaccharide. During plasma exposure, ROS can react with peptidoglycan and lipopolysaccharide and, by breaking down the C‐O, C‐N, and C‐C bonds, damage the molecular structure of these materials and destroyed them (Han et al., 2016). It should be noted that increased plasma exposure time causes more bacteria to be damaged and, in many cases, many deformities occur in their bacterial cell membrane and appearance (Ali et al., 2014; Han et al., 2016; Yang et al., 2011).

### 3.4 The effect of plasma on the qualitative properties of sour cherry juice

The ANOVA results regarding the effect of APCP on the TPC, TAC, and vitamin C are shown in Table 3, and the obtained models for TAC in the encoded and actual conditions are presented in Equations 12 and 13 and for vitamin C in Equations 14 and 15. The explanation coefficient and standard error of the models were 0.967 and 0.70 for TAC and 0.972 and 0.51 for vitamin C, respectively. Table 3 shows the effects of plasma exposure time, depth of samples, field intensity, and oxygen content in argon on the TPC of the sour cherry juice samples were not significant (*p* > .01) but those on the TAC and vitamin C of the juice were significant (*p* < .01).

(12) $$\begin{array}{c} \text{TAC}\;\left(\text{mg C}3\text{GE}/L\right)=238.21-1.46\times \text{Time}+0.96\times \text{Depth}-1.39\times \text{Field Strength}-2.15\times \text{Oxygen in Argon}\\ +0.004\times \left(\text{Time}\times \;\text{Depth}\right)-0.53\times \left(\text{Time}\times \text{Field Strength}\right)-0.14\times \left(\text{Time}\times \text{Oxygen in Argon}\right)\\ -0.10\times \left(\text{Depth}\times \text{Field Strength}\right)+0.22\times \left(\text{Depth}\times \text{Oxygen in Argon}\right)+0.03\times \left(\text{Field Strength}\times \;\text{Oxygen in Argon}\right)\\ +0.16{\left(\text{Time}\right)}^{2}-0.85\times {\left(\text{Depth}\right)}^{2}-0.20\times {\left(\text{Field Strength}\right)}^{2}-1.39\times {\left(\text{Oxygen in Argon}\right)}^{2} \end{array}$$

(13) $$\begin{array}{c} \text{TAC}\;\left(\text{mg C}3\text{GE}/L\right)=235.85-0.03\times \text{Time}+8.86\times \text{Depth}+0.05\times \text{Field Strength}+0.54\times \text{Oxygen in Argon}\\ -0.002\times \left(\text{Time}\times \;\text{Depth}\right)-0.01\times \left(\text{Time}\times \text{Field Strength}\right)-0.07\times \left(\text{Time}\times \text{Oxygen in Argon}\right)\\ -0.02\times \left(\text{Depth}\times \text{Field Strength}\right)+0.89\times \left(\text{Depth}\times \text{Oxygen in Argon}\right)-0.005\times \left(\text{Field Strength}\times \;\text{Oxygen in Argon}\right)\\ -0.01\times {\left(\text{Time}\right)}^{2}-2.81\times {\left(\text{Depth}\right)}^{2}-0.001\times {\left(\text{Field Strength}\right)}^{2}-5.58\times {\left(\text{Oxygen in Argon}\right)}^{2} \end{array}$$

(14) $$\begin{array}{c} \text{Vitamin C}\;\left(\text{mg}/L\right)=33.82-1.02\times \text{Time}+0.66\times \text{Depth}-0.53\times \text{Field Strength}-2.24\times \text{Oxygen in Argon}\\ -0.19\times \left(\text{Time}\times \;\text{Depth}\right)-0.14\times \left(\text{Time}\times \text{Field Strength}\right)-0.21\times \left(\text{Time}\times \text{Oxygen in Argon}\right)\\ +0.24\times \left(\text{Depth}\times \text{Field Strength}\right)+0.07\times \left(\text{Depth}\times \text{Oxygen in Argon}\right)-0.01\times \left(\text{Field Strength}\times \;\text{Oxygen in Argon}\right)\\ -0.23\times {\left(\text{Time}\right)}^{2}-0.70\times {\left(\text{Depth}\right)}^{2}-0.27\times {\left(\text{Field Strength}\right)}^{2}-0.42\times {\left(\text{Oxygen in Argon}\right)}^{2} \end{array}$$

(15) $$\begin{array}{c} \text{Vitamin C}\;\left(\text{mg}/L\right)=31.80+0.14\times \text{Time}+5.86\times \text{Depth}+0.08\times \text{Field Strength}-2.49\times \text{Oxygen in Argon}\\ -0.09\times \left(\text{Time}\times \;\text{Depth}\right)-0.002\times \left(\text{Time}\times \text{Field Strength}\right)-0.10\times \left(\text{Time}\times \text{Oxygen in Argon}\right)\\ +0.04\times \left(\text{Depth}\times \text{Field Strength}\right)+0.27\times \left(\text{Depth}\times \text{Oxygen in Argon}\right)-0.002\times \left(\text{Field Strength}\times \;\text{Oxygen in Argon}\right)\\ -0.01\times {\left(\text{Time}\right)}^{2}-2.81\times {\left(\text{Depth}\right)}^{2}-0.002\times {\left(\text{Field Strength}\right)}^{2}-1.68\times {\left(\text{Oxygen in Argon}\right)}^{2} \end{array}$$

**Table 3:** The analysis of variance results for the effect of ACPC on qualitative properties (TPC, TAC and vitamin C) in sour cherry juice

Source	*df*	TPC (mg GAE/100 g)		TA (mg C3GE/L)		Vitamin C (mg/L)	
		Sum of squares	Mean square	Sum of squares	Mean square	Sum of squares	Mean square
Model	14	20.25	1.45^ns^	211.69	15.12 **	140.02	10.00 **
Time (min)	1	3.19	3.19^ns^	38.49	38.49 **	18.83	18.83 **
Depth (cm)	1	1.21	1.21^ns^	16.59	16.59 **	7.75	7.75 **
Field strength (kV/cm)	1	0.88	0.88^ns^	34.72	34.72 **	4.96	4.96 **
Oxygen in argon (%)	1	1.03	1.03^ns^	83.21	83.21**	90.63	90.63 **
Time × depth	1	1.12	1.12^ns^	2.250E‐004	2.25E‐004^ns^	0.56	0.56^ns^
Time × field strength	1	2.85	2.85^ns^	4.54	4.54**	0.32	0.32^ns^
Time × oxygen in argon	1	0.24	0.24^ns^	0.32	0.32^ns^	0.69	0.69^ns^
Depth × field strength	1	0.077	0.077^ns^	0.14	0.14^ns^	0.89	0.89^ns^
Depth × oxygen in argon	1	0.079	0.079^ns^	0.79	0.79^ns^	0.074	0.074^ns^
Field strength × oxygen in argon	1	3.02	3.02^ns^	0.018	0.018^ns^	2.162E‐003	2.162E‐003^ns^
Time^2^	1	1.57	1.57^ns^	0.063	0.063^ns^	0.13	0.13^ns^
Depth^2^	1	0.012	0.012^ns^	1.89	1.89^ns^	1.28	1.28^ns^
Field strength^2^	1	0.71	0.71^ns^	0.11	0.11^ns^	0.20	0.20^ns^
Oxygen in argon^2^	1	0.53	0.53^ns^	5.03	5.03**	0.46	0.46^ns^
Residual	15	10.23	0.68	7.28	0.49	3.96	0.26
Lack of fit	10	7.21	0.72^ns^	5.03	0.50^ns^	2.44	0.24^ns^
Pure error	5	3.02	0.60	2.25	0.45	1.52	0.30
Cor total	29	30.48		218.97		143.98	

Note

^ns^Not significant.

** Significant Effect at 1% level.

The TPC of the untreated sour cherry juice samples was calculated at 277.05 ± 2.1 mg GAE/100 g and that of the treated samples ranged from 273.01 ± 1.07 to 276.28 ± 0.91 mg GAE/100 g. These results indicated that the TPC in the sour cherry juice remained unchanged after plasma exposure (Table 3). This result is consistent with the results of a study that investigated the effect of cold plasma on the chemical properties of strawberries (Misra, Pankaj, Frias, Keener, & Cullen, 2015). However, researches' results in this regard are contradictory. The results of some studies indicate that plasma exposure increases the TPC in pomegranate juice (Herceg et al., 2016) and sour cherry juice (Garofulić et al., 2015). In addition, plasma exposure reduced the phenolic compounds of the lettuce leaf samples (Grzegorzewski, Ehlbeck, Schlüter, Kroh, & Rohn, 2011), but we observed no change in the TPC of the sour cherry juice due to plasma exposure in the current study (*p* > .01).

**Figure 6:**
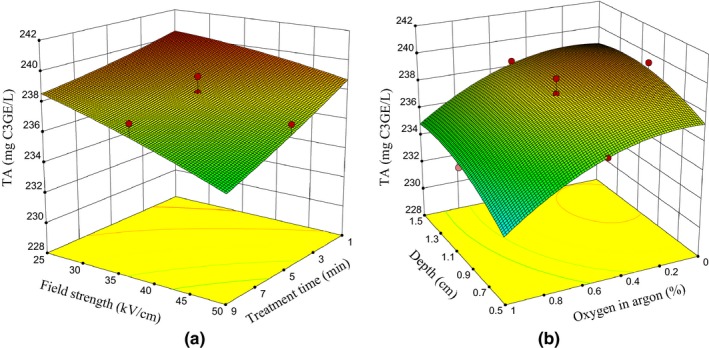
(a) The effect of field intensity and plasma exposure time, and (b) the effect of samples depth and oxygen content in argon on TAC in sour cherry juice

Table 3 shows that plasma exposure alters the TAC of the sour cherry juice. The TAC of untreated samples, and the lowest and highest TACs of treated samples were 241.20 ± 0.32, 240.70 ± 2.6, and 229.80 ± 1.41 mg C3GE/L, respectively. In other words, the greatest decrease in TAC of sample is 7.4 percent per liter. According to Equation 12, by increasing the plasma exposure time and field intensity (Figure 6a), as well as by increasing the oxygen percentage in argon, and decreasing the depth of the samples (Figure 6b), TAC of sour cherry juice was decreased. Several factors such as heat, light, and oxygen radicals can cause denaturation of anthocyanin compounds (Lacombe et al., 2015).

Since the temperature of treated samples in this study was up to 21°C and is lower than the temperature required for breakdown of anthocyanin, that is, 38°C (Tiwari, Patras, Brunton, Cullen, & O'Donnell, 2010), the effect of this factor seems negligible, and the effect of oxygen and ozone radicals was considered as the most important anthocyanin reduction factor (Figure 3; Lacombe et al., 2015; Tiwari, O'Donnell, & Cullen, 2009; Tiwari et al., 2010). Confirmation of this is that by increasing ROS‐enhancing agents, the anthocyanin in the sour cherry juice was a further reduction (Figure 6a,b).

The results of studies on the effect of plasma on the TAC are also inconsistent. The study of Lacombe et al. (2015) illustrates that being prone to plasma for 90 s lead to a significant reduction in the anthocyanin content of the blueberry samples (Lacombe et al., 2015). Likewise, the research has been carried out by Tiwari, O'Donnell, and Cullen (2009) and Tiwari, O'Donnell, Patras, Brunton, and Cullen (2009) and reveals ozone could reduce more than 90% of the anthocyanin present in the blackberry juice. However, some studies have shown plasma exposure increases the anthocyanin content of the sour cherry juice by 15% (Garofulić et al., 2015) and increases the anthocyanin content of pomegranate juice (Kovačević et al., 2016). Misra et al. (2015) reported plasma had no effect on the anthocyanin content of strawberries.

**Figure 7:**
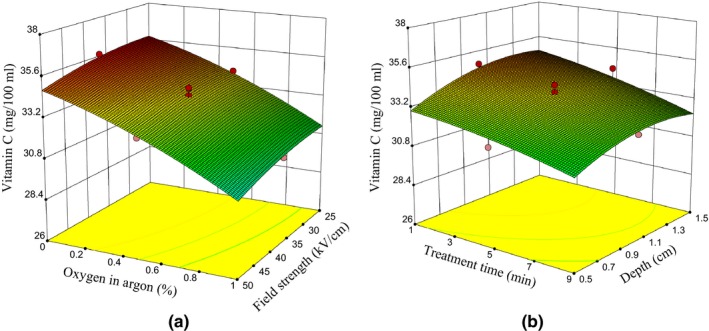
(a) The effect of oxygen percentage in argon, field intensity, and (b) the effect of the depth of samples and plasma exposure time on vitamin C range in sour cherry juice

Vitamin C of untreated samples was calculated at 36.03 ± 1.24 mg/L, but that of plasma‐treated samples varied from 27.92 ± 0.9 to 5.79 ± 2.20 mg/L. The ANOVA results (Table 3) show plasma has a significant effect on vitamin C in the sour cherry juice samples (*p* < .01). According to Equation 14 and Table 3, it can be inferred that increasing the oxygen content in argon, in comparison with other variables, explains a higher percentage of variance in vitamin C of the samples. Therefore, the presence of molecules and oxygen radicals can be considered as the main cause of the reduction in vitamin C of the sour cherry juice. These particles lead to oxidation of vitamin C (Misra et al., 2015; Shi et al., 2011; Wang et al., 2012). The results of our study show that increasing oxygen content, increasing field intensity (Figure 7a), increasing plasma exposure time, and decreasing the depth of samples (Figure 7a), reduce the amount of vitamin C. The increase in plasma exposure time and applied voltage (directly correlated with field intensity) has been reported to reduce the vitamin C of the strawberry samples (Misra et al., 2015). Another study reported that plasma reduced vitamin C of pieces of cucumber, hawthorn, and pear by 3.6, 3.2, and 2.8, respectively (Wang et al., 2012). Also, in another study, ozone reduced vitamin C of strawberries by 50% within approximately 5 min (Tiwari, O'Donnell, Patras, et al., 2009). While, exposing DBD‐plasma generated by air to orange juice for 5, 10, 15, and 20 s showed that plasma has no significant effect on vitamin C in the samples (*p* > .05; Shi et al., 2011).

### 3.5 Optimizing the results and comparing with the results of the conventional thermal method

In this section, the optimal conditions for use of APCP were determined in two states: state 1: The time when all *E. coli* bacteria were destroyed and changes in qualitative properties were determined when the maximum reduction in bacterial number occurred and state 2: The time when the least changes in the qualitative properties of the sour cherry juice occurred and the maximum reduction in bacterial number was determined according to these conditions. Then, the results obtained under optimal conditions were compared with those obtained through thermal methods. The boundary conditions of optimization are shown in Table 4. The results of the optimization in two states are shown in Table 5.

**Table 4:** Boundary conditions used for the optimization

Name	Goal (State 1)	Goal (State 2)	Lower limit	Upper limit	Lower weight	Upper weight	Importance
Time (min)	In range	In range	1	9	1	1	3
Depth (cm)	In range	In range	0.5	1.5	1	1	3
Field strength (kV/cm)	In range	In range	25	50	1	1	3
Oxygen in argon (%)	In range	In range	0	1	1	1	3
Log (*N*/*N*_0_)	Minimize	Minimize	−5.994	−0.222	1	1	3
TPC (mg GAE/100 g)	In range	Maximize	273.02	276.23	1	1	3
TAC (mg C3GE/L)	In range	Maximize	229.80	240.70	1	1	3
Vitamin C (mg/L)	In range	Maximize	37.92	45.79	1	1	3

**Table 5:** Optimization results using the RSM method

Sour cherry juice samples	Results				Variation rather than untreated samples			
	*E. coli*	TAC	TPC	Vitamin C	*E. coli*	TAC	TPC	Vitamin C
Untreated	5.994	241.20 ± 1.3a	277.05 ± 1.20b	36.03 ± 1.24a	0	0	0	0
Thermal treated	0	171.5 ± 0.49d	359.75 ± 2.07a	8.23 ± 0.32c	100% (↓)	26% (↓)	23% (↑)	77% (↓)
Plasma treated								
State 1	0	230.52 ± 1.12c	275.04 ± 2.27b	28.29 ± 1.06b	100% (↓)	4% (↓)	0	21% (↓)
Plasma treated								
State 2	2.25	238.87 ± 1.19b	274.98 ± 2.97b	34.12 ± 1.25a	38% (↓)	0%	0	5% (↓)

Note

*Escherichia coli* is expressed in terms of (CFU/ml), TAC in terms of (mg C3GE/L), TPC in terms of mg GAE/100 g, and vitamin C in terms of (mg/L). Different letters showed significant differences at 5% Duncan test.

The results of optimization show that under the boundary conditions (Table 4), the optimum exposure time, field intensity, oxygen percentage, and depth of samples were 8.8 min, 49 kV/cm, 1%, and 0.65 cm in two statuses 1 and 1 min, 50 kV/cm, 0.5% and 1.1 cm in two statuses 2, respectively. Table 5 shows the variation of qualitative properties of the sour cherry juice in states 1 and 2 as well. In this Table, the effects of thermal methods on the sour cherry juice also shown. As Table 5 shows, under optimal conditions in the state 1, plasma does not change in the TPC of samples and reduces their TAC and vitamin C content by 4% and 21%, respectively (Kovačević et al., 2016; Misra et al., 2015; Wang et al., 2012). The decomposition of anthocyanins and ascorbic acid can be due to oxidative reactions caused by free radicals that are appeared in the plasma generation process. The greater decomposition of ascorbic acid, in comparison with anthocyanins, is due to its physical and chemical characteristics, because ascorbic acid is much more vulnerable against free radicals (Hosseinzadeh Samani et al., 2015).

Table 5 also shows that in state 2, with maximum preservation of the qualitative properties of sour cherry juice, 38% of *E. coli* bacteria in the sour cherry juice were destroyed. In this state, there was no change in the TAC and TPC, but the vitamin C decreased by 5%. While in conventional thermal methods, the TPC of the sour cherry juice has increased due to exposure to high temperatures (above 80°C; Garofulić et al., 2015; Herceg et al., 2016). In this study, the increase in TPC in the thermal method is 26%. Studies have shown that phenolic compounds are formed at temperatures above 80–90°C, due to the supply of phenolic compounds' precursors as well as nonenzymatic exchanges between the molecules. In fact, in this temperature due to the inactivation of enzymes that are effective on decomposition and degradation, new phenolic compounds are formed. Also, pasteurization by conventional thermal method reduced the TAC and vitamin C content by 23% and 77%, respectively. The reduction of vitamin C and TAC by the thermal method has already been confirmed (Samani et al., 2016). These results show that in comparison with the thermal methods, the pasteurization of the sour cherry juice by the APCP can help to maintain its qualitative properties. This result is consistent with the results of studies that have investigated plasma's effects on the qualitative properties of the products (Gurol et al., 2012; Misra, Keener, Bourke, Mosnier, & Cullen, 2014; Shi et al., 2011).

## 4 CONCLUSION

In this study, the effects of the APCP on inactivation of *E. coli* bacteria in sour cherry juice and the qualitative properties of the juice were investigated. The results of this study indicate that by increasing the plasma exposure time, the field intensity, and the percentage of oxygen, and reducing the depth of samples, more *E. coli* bacteria were destroyed, and among these variables, increase of oxygen content plays the most substantial role due to the production of more ROS. The results also showed plasma had no effect on the TPC of the samples and, compared to the thermal methods, had a small effect on the TAC and ascorbic acid in sour cherry juice. This method can, therefore, be safely used to eliminate the bacteria without influencing the qualitative properties of the materials.

## CONFLICT OF INTEREST

The authors declare that they do not have any conflict of interest.

## ETHICAL STATEMENT

This study does not involve any human or animal testing.

## References

[fsn31364-bib-0001] Adekunte, A. , Tiwari, B. , Cullen, P. , Scannell, A. , & O'Donnell, C. (2010). Effect of sonication on colour, ascorbic acid and yeast inactivation in tomato juice. Food Chemistry, 122(3), 500–507. 10.1016/j.foodchem.2010.01.026

[fsn31364-bib-0002] Ali, A. , Kim, Y. H. , Lee, J. Y. , Lee, S. H. , Uhm, H. S. , Cho, G. , … Choi, E. H. (2014). Inactivation of Propionibacterium acnes and its biofilm by non‐thermal plasma. Current Applied Physics, 14, S142–S148. 10.1016/j.cap.2013.12.034

[fsn31364-bib-0003] Anonymous (2018). Determination of vitamin C concentration by titration. Available from: http://www.canterbury.ac.nz/media/documents/science-outreach/vitaminc_iodine.pdf. Accessed December 22, 2018.

[fsn31364-bib-0004] Behruzian, A. , Hosseinzadeh Samani, B. , Rostami, S. , Lorigooini, Z. , & Behruzian, M. (2018). The effect of combined AC electric field and ultrasound on the chemical compositions and *Escherichia coli* content of spearmint aromatic water. Journal of Food Process Engineering, 41, e12650.

[fsn31364-bib-0005] Bermúdez‐Aguirre, D. , Wemlinger, E. , Pedrow, P. , Barbosa‐Cánovas, G. , & Garcia‐Perez, M. (2013). Effect of atmospheric pressure cold plasma (APCP) on the inactivation of *Escherichia coli* in fresh produce. Food Control, 34(1), 149–157. 10.1016/j.foodcont.2013.04.022

[fsn31364-bib-0006] Blando, F. , Gerardi, C. , & Nicoletti, I. (2004). Sour cherry (*Prunus cerasus* L) anthocyanins as ingredients for functional foods. BioMed Research International, 2004(5), 253–258. 10.1155/S1110724304404136 PMC1082898 15577186

[fsn31364-bib-0007] Bruggeman, P. J. , Kushner, M. J. , Locke, B. R. , Gardeniers, J. G. E. , Graham, W. G. , Graves, D. B. , … Zvereva, G. (2016). Plasma–liquid interactions: A review and roadmap. Plasma Sources Science Technology, 25(5), 59 10.1088/0963-0252/25/5/053002

[fsn31364-bib-0008] Coutinho, N. M. , Silveira, M. R. , Fernandes, L. M. , Moraes, J. , Pimentel, T. C. , Freitas, M. Q. , … Cruz, A. G. (2019). Processing chocolate milk drink by low‐pressure cold plasma technology. Food Chemistry, 278, 276–283. 10.1016/j.foodchem.2018.11.061 30583374

[fsn31364-bib-0009] Deng, S. , Cheng, C. , Ni, G. , Meng, Y. , & Chen, H. (2010). Bacillus subtilis devitalization mechanism of atmosphere pressure plasma jet. Current Applied Physics, 10(4), 1164–1168. 10.1016/j.cap.2010.02.004

[fsn31364-bib-0010] Deng, X. , Shi, J. , & Kong, M. G. (2006). Physical mechanisms of inactivation of Bacillus subtilis spores using cold atmospheric plasmas. IEEE Transactions on Plasma Science, 34, 1310–1316. 10.1109/TPS.2006.877739

[fsn31364-bib-0011] Fridman, A. , & Kennedy, L. A. (2004). Plasma physics and engineering. Boca Raton, FL: CRC Press.

[fsn31364-bib-0012] Fröhling, A. , Durek, J. , Schnabel, U. , Ehlbeck, J. , Bolling, J. , & Schlüter, O. (2012). Indirect plasma treatment of fresh pork: Decontamination efficiency and effects on quality attributes. Innovative Food Science Emerging Technologies, 16, 381–390. 10.1016/j.ifset.2012.09.001

[fsn31364-bib-0013] Gao, Y. , Zhuang, H. , Yeh, H.‐Y. , Bowker, B. , & Zhang, J. (2019). Effect of rosemary extract on microbial growth, pH, color, and lipid oxidation in cold plasma‐processed ground chicken patties. Innovative Food Science & Emerging Technologies, 57, 102168 10.1016/j.ifset.2019.05.007

[fsn31364-bib-0014] Garofulić, I. E. , Jambrak, A. R. , Milošević, S. , Dragović‐Uzelac, V. , Zorić, Z. , & Herceg, Z. (2015). The effect of gas phase plasma treatment on the anthocyanin and phenolic acid content of sour cherry Marasca (*Prunus cerasus* var. Marasca) juice. LWT‐Food Science Technology, 62(1), 894–900. 10.1016/j.lwt.2014.08.036

[fsn31364-bib-0015] Grzegorzewski, F. , Ehlbeck, J. , Schlüter, O. , Kroh, L. W. , & Rohn, S. (2011). Treating lamb's lettuce with a cold plasma–Influence of atmospheric pressure Ar plasma immanent species on the phenolic profile of *Valerianella locusta* . LWT‐Food Science Technology, 44(10), 2285–2289. 10.1016/j.lwt.2011.05.004

[fsn31364-bib-0016] Gurol, C. , Ekinci, F. , Aslan, N. , & Korachi, M. (2012). Low temperature plasma for decontamination of *E. coli* in milk. International Journal of Food Microbiology, 157(1), 1–5. 10.1016/j.ijfoodmicro.2012.02.016 22622128

[fsn31364-bib-0017] Han, L. , Patil, S. , Boehm, D. , Milosavljević, V. , Cullen, P. , & Bourke, P. (2016). Mechanisms of inactivation by high‐voltage atmospheric cold plasma differ for *Escherichia coli* and *Staphylococcus aureus* . Applied Environmental Microbiology, 82(2), 450–458. 10.1128/AEM.02660-15 26519396 PMC4711144

[fsn31364-bib-0018] Harouni, A. , & Abbasi, S. (2013). Designing a cold plasma generator and its effect on decontamination of inocualated egg shells and aluminum foil. Paper presented at the 1st Electronic Conference on Innovations in food processing, Ferdowsi University of Mashhad, Mashhad.

[fsn31364-bib-0019] Herceg, Z. , Kovačević, D. B. , Kljusurić, J. G. , Jambrak, A. R. , Zorić, Z. , & Dragović‐Uzelac, V. (2016). Gas phase plasma impact on phenolic compounds in pomegranate juice. Food Chemistry, 190, 665–672. 10.1016/j.foodchem.2015.05.135 26213024

[fsn31364-bib-0020] Hosseinzadeh Samani, B. , Minaee, S. , & Khoshtaghaza, M. (2015). Modeling the simultaneous effects of microwave and ultrasound treatments on sour cherry juice using response surface methodology. Journal of Agricultural Science and Technology, 17, 837–846.

[fsn31364-bib-0021] Hou, Y. , Wang, R. , Gan, Z. , Shao, T. , Zhang, X. , He, M. , & Sun, A. (2019). Effect of cold plasma on blueberry juice quality. Food Chemistry, 290, 79–86. 10.1016/j.foodchem.2019.03.123 31000059

[fsn31364-bib-0022] Huang, C. , Yu, Q. , Hsieh, F. H. , & Duan, Y. (2007). Bacterial deactivation using a low temperature argon atmospheric plasma brush with oxygen addition. Plasma Processes Polymers, 4(1), 77–87. 10.1002/ppap.200600077

[fsn31364-bib-0023] Kovačević, D. B. , Putnik, P. , Dragović‐Uzelac, V. , Pedisić, S. , Jambrak, A. R. , & Herceg, Z. (2016). Effects of cold atmospheric gas phase plasma on anthocyanins and color in pomegranate juice. Food Chemistry, 190, 317–323. 10.1016/j.foodchem.2015.05.099 26212976

[fsn31364-bib-0024] Lacombe, A. , Niemira, B. A. , Gurtler, J. B. , Fan, X. , Sites, J. , Boyd, G. , & Chen, H. (2015). Atmospheric cold plasma inactivation of aerobic microorganisms on blueberries and effects on quality attributes. Food Microbiology, 46, 479–484. 10.1016/j.fm.2014.09.010 25475318

[fsn31364-bib-0025] Laroussi, M. , & Leipold, F. (2004). Evaluation of the roles of reactive species, heat, and UV radiation in the inactivation of bacterial cells by air plasmas at atmospheric pressure. International Journal of Mass Spectrometry, 233(1–3), 81–86. 10.1016/j.ijms.2003.11.016

[fsn31364-bib-0026] Lee, J. , Durst, R. W. , & Wrolstad, R. E. (2005). Determination of total monomeric anthocyanin pigment content of fruit juices, beverages, natural colorants, and wines by the pH differential method: Collaborative study. Journal of AOAC International, 88, 1269–1278. 16385975

[fsn31364-bib-0027] Li, J. , Sakai, N. , Watanabe, M. , Hotta, E. , & Wachi, M. (2013). Study on plasma agent effect of a direct‐current atmospheric pressure oxygen‐plasma jet on inactivation of *E. coli* using bacterial mutants. IEEE Transactions on Plasma Science, 41(4), 935–941. 10.1109/TPS.2013.2248395

[fsn31364-bib-0028] Liang, T. , Yue, W. , & Li, Q. (2010). Comparison of the phenolic content and antioxidant activities of *Apocynum venetum* L. (Luo‐Bu‐Ma) and two of its alternative species. International Journal of Molecular Sciences, 11(11), 4452–4464. 21151449 10.3390/ijms11114452 PMC3000093

[fsn31364-bib-0029] Lu, H. , Patil, S. , Keener, K. M. , Cullen, P. , & Bourke, P. (2014). Bacterial inactivation by high‐voltage atmospheric cold plasma: Influence of process parameters and effects on cell leakage and DNA. Journal of Applied Microbiology, 116(4), 784–794. 10.1111/jam.12426 24372804

[fsn31364-bib-0030] Misra, N. N. , Keener, K. M. , Bourke, P. , Mosnier, J.‐P. , & Cullen, P. J. (2014). In‐package atmospheric pressure cold plasma treatment of cherry tomatoes. Journal of Bioscience Bioengineering, 118(2), 177–182. 10.1016/j.jbiosc.2014.02.005 24650730

[fsn31364-bib-0031] Misra, N. , Pankaj, S. , Frias, J. , Keener, K. , & Cullen, P. (2015). The effects of nonthermal plasma on chemical quality of strawberries. Postharvest Biology Technology, 110, 197–202. 10.1016/j.postharvbio.2015.08.023

[fsn31364-bib-0032] Misra, N. , Schlüter, O. , & Cullen, P. J. (2016). Cold plasma in food and agriculture: Fundamentals and applications. Cambridge, MA: Academic Press.

[fsn31364-bib-0033] Mohamed, A.‐A.‐H. , Al Shariff, S. M. , Ouf, S. A. , & Benghanem, M. (2016). Atmospheric pressure plasma jet for bacterial decontamination and property improvement of fruit and vegetable processing wastewater. Journal of Physics D: Applied Physics, 49(19), 195401 10.1088/0022-3727/49/19/195401

[fsn31364-bib-0034] Mohamed, M. E. , & Eissa, A. H. A. (2012). Pulsed electric fields for food processing technology In Eissa A. H. A. (Ed.) Structure and function of food engineering (pp. 275–307). Rijeka, Croatia: InTech.

[fsn31364-bib-0035] Niemira, B. A. (2012). Cold plasma decontamination of foods. Annual Review of Food Science Technology, 3, 125–142. 10.1146/annurev-food-022811-101132 22149075

[fsn31364-bib-0036] Niemira, B. A. , Boyd, G. , & Sites, J. (2018). Cold plasma inactivation of *Escherichia coli* O157: H7 biofilms. Frontiers in Sustainable Food Systems, 2, 47 10.3389/fsufs.2018.00047

[fsn31364-bib-0037] Noriega, E. , Shama, G. , Laca, A. , Díaz, M. , & Kong, M. G. (2011). Cold atmospheric gas plasma disinfection of chicken meat and chicken skin contaminated with *Listeria innocua* . Food Microbiology, 28(7), 1293–1300. 10.1016/j.fm.2011.05.007 21839378

[fsn31364-bib-0038] Pei, X. , Lu, X. , Liu, J. , Liu, D. , Yang, Y. , Ostrikov, K. , … Pan, Y. (2012). Inactivation of a 25.5 µm *Enterococcus faecalis* biofilm by a room‐temperature, battery‐operated, handheld air plasma jet. Journal of Physics D: Applied Physics, 45(16), 165205.

[fsn31364-bib-0039] Perni, S. , Liu, D. W. , Shama, G. , & Kong, M. G. (2008). Cold atmospheric plasma decontamination of the pericarps of fruit. Journal of Food Protection, 71(2), 302–308. 10.4315/0362-028X-71.2.302 18326179

[fsn31364-bib-0040] Ragni, L. , Berardinelli, A. , Vannini, L. , Montanari, C. , Sirri, F. , Guerzoni, M. E. , & Guarnieri, A. (2010). Non‐thermal atmospheric gas plasma device for surface decontamination of shell eggs. Journal of Food Engineering, 100(1), 125–132. 10.1016/j.jfoodeng.2010.03.036

[fsn31364-bib-0041] Rouhani, R. , Eyenafshar, S. , & Ahmadzadeh, R. (2015). Study of anthocyanin and antionidant compounds derived ethanol extract saffron flag with the help of ultrasound technology. Iranian Food Science and Technology Research Journal, 11, 161–170.

[fsn31364-bib-0042] Samani, B. H. , Khoshtaghaza, M. H. , Minaei, S. , Zareifourosh, H. , Eshtiaghi, M. N. , & Rostami, S. (2016). Design, development and evaluation of an automatic fruit‐juice pasteurization system using microwave–ultrasonic waves. Journal of Food Science Technology, 53(1), 88–103. 10.1007/s13197-015-2026-6 26787934 PMC4711452

[fsn31364-bib-0043] Samani, B. H. , Lorigooini, Z. , Rostami, S. , Zareiforoush, H. , Behruzian, M. , & Behruzian, A. (2018). The simultaneous effect of electromagnetic and ultrasound treatments on *Escherichia coli* count in red grape juice. Journal of Herbmed Pharmacology, 7(1), 29–36.

[fsn31364-bib-0044] Samani, H. B. , Gudarzi, H. , Rostami, S. , Lorigooini, Z. , Esmaeili, Z. , & Jamshidi‐kia, F. (2018). Development and optimization of the new ultrasonic‐infrared‐vacuum dryer in drying *Kelussia odoratissima* and its comparison with conventional methods. Industrial Crops and Products, 123, 46–54. 10.1016/j.indcrop.2018.06.053

[fsn31364-bib-0045] Segura‐Ponce, L. A. , Reyes, J. E. , Troncoso‐Contreras, G. , & Valenzuela‐Tapia, G. (2018). Effect of low‐pressure cold plasma (LPCP) on the wettability and the inactivation of *Escherichia coli* and *Listeria innocua* on fresh‐cut apple (Granny Smith) skin. Food Bioprocess Technology, 11(5), 1075–1086. 10.1007/s11947-018-2079-4

[fsn31364-bib-0046] Shi, X.‐M. , Zhang, G.‐J. , Wu, X.‐L. , Li, Y.‐X. , Ma, Y. , & Shao, X.‐J. (2011). Effect of low‐temperature plasma on microorganism inactivation and quality of freshly squeezed orange juice. IEEE Transactions on Plasma Science, 39(7), 1591–1597. 10.1109/TPS.2011.2142012

[fsn31364-bib-0047] Song, H. P. , Kim, B. , Choe, J. H. , Jung, S. , Moon, S. Y. , Choe, W. , & Jo, C. (2009). Evaluation of atmospheric pressure plasma to improve the safety of sliced cheese and ham inoculated by 3‐strain cocktail *Listeria monocytogenes* . Food Microbiology, 26(4), 432–436. 10.1016/j.fm.2009.02.010 19376467

[fsn31364-bib-0048] Surowsky, B. , Fröhling, A. , Gottschalk, N. , Schlüter, O. , & Knorr, D. (2014). Impact of cold plasma on *Citrobacter freundii* in apple juice: Inactivation kinetics and mechanisms. International Journal of Food Microbiology, 174, 63–71. 10.1016/j.ijfoodmicro.2013.12.031 24462703

[fsn31364-bib-0049] Tiwari, B. K. , O'Donnell, C. P. , & Cullen, P. J. (2009). Effect of non thermal processing technologies on the anthocyanin content of fruit juices. Trends in Food Science Technology, 20(3–4), 137–145. 10.1016/j.tifs.2009.01.058

[fsn31364-bib-0050] Tiwari, B. K. , O'Donnell, C. P. , Patras, A. , Brunton, N. , & Cullen, P. J. (2009). Effect of ozone processing on anthocyanins and ascorbic acid degradation of strawberry juice. Food Chemistry, 113(4), 1119–1126. 10.1016/j.foodchem.2008.08.085

[fsn31364-bib-0051] Tiwari, B. , Patras, A. , Brunton, N. , Cullen, P. , & O'Donnell, C. (2010). Effect of ultrasound processing on anthocyanins and color of red grape juice. Ultrasonics Sonochemistry, 17(3), 598–604. 10.1016/j.ultsonch.2009.10.009 20015673

[fsn31364-bib-0052] Tolouie, H. , Mohammadifar, M. A. , Ghomi, H. , & Hashemi, M. (2017). Cold atmospheric plasma manipulation of proteins in food systems. Critical Reviews in Food Science Nutrition, 58, 2583–2597. 28613926 10.1080/10408398.2017.1335689

[fsn31364-bib-0053] Toydemir, G. , Capanoglu, E. , Roldan, M. V. G. , de Vos, R. C. , Boyacioglu, D. , Hall, R. D. , & Beekwilder, J. (2013). Industrial processing effects on phenolic compounds in sour cherry (*Prunus cerasus* L.) fruit. Food Research International, 53(1), 218–225. 10.1016/j.foodres.2013.04.009

[fsn31364-bib-0054] Uhm, H. S. , Lim, J. P. , & Li, S. Z. (2007). Sterilization of bacterial endospores by an atmospheric‐pressure argon plasma jet. Applied Physics Letters, 90(26), 261501 10.1063/1.2747177

[fsn31364-bib-0055] Wang, R. X. , Nian, W. F. , Wu, H. Y. , Feng, H. Q. , Zhang, K. , Zhang, J. , … Fang, J. (2012). Atmospheric‐pressure cold plasma treatment of contaminated fresh fruit and vegetable slices: Inactivation and physiochemical properties evaluation. The European Physical Journal D, 66(10), 276 10.1140/epjd/e2012-30053-1

[fsn31364-bib-0056] Xiaohu, L. , Feng, H. , Ying, G. , Jing, Z. , & Jianjun, S. (2013). Sterilization of *Staphylococcus aureus* by an atmospheric non‐thermal plasma jet. Plasma Science Technology, 15(5), 439 10.1088/1009-0630/15/5/09

[fsn31364-bib-0057] Xiong, Z. , Du, T. , Lu, X. , Cao, Y. , & Pan, Y. (2011). How deep can plasma penetrate into a biofilm? Applied Physics Letters, 98(22), 221503 10.1063/1.3597622

[fsn31364-bib-0058] Yang, B. O. , Chen, J. , Yu, Q. , Li, H. , Lin, M. , Mustapha, A. , … Wang, Y. (2011). Oral bacterial deactivation using a low‐temperature atmospheric argon plasma brush. Journal of Dentistry, 39(1), 48–56. 10.1016/j.jdent.2010.10.002 20951184 PMC3010533

[fsn31364-bib-0059] Ziuzina, D. , Patil, S. , Cullen, P. J. , Keener, K. , & Bourke, P. (2014). Atmospheric cold plasma inactivation of *Escherichia coli*, *Salmonella enterica* serovar Typhimurium and *Listeria monocytogenes* inoculated on fresh produce. Food Microbiology, 42, 109–116. 10.1016/j.fm.2014.02.007 24929725

